# Huntington's Disease Gene Hunters: An Expanding Tale

**DOI:** 10.1002/mdc3.13375

**Published:** 2021-12-16

**Authors:** Anne E. Rosser, Lesley Jones

**Affiliations:** ^1^ MRC Centre for Neuropsychiatric Genetics and Genomics, Division of Psychological Medicine and Clinical Neurosciences Cardiff University School of Medicine Cardiff United Kingdom; ^2^ Cardiff Brain Repair Group Cardiff University School Biosciences Cardiff United Kingdom; ^3^ Wales Brain Research And Intracranial Neurotherapeutics (BRAIN) Unit Cardiff United Kingdom

**Keywords:** Huntington's, gene discovery, CAG repeat expansion

MacDonald ME. *A novel gene containing a trinucleotide repeat that is expanded and unstable on Huntington's disease chromosomes*. Cell 1993;72:971–983.10.1016/0092-8674(93)90585-e8458085

It is 28 years since the Huntington's Disease (HD) gene and mutation were identified and published in *Cell* by the Huntington's Disease Collaborative Research Group (HD‐CRG; Fig. 1A).^1^ The genetic defect causing HD had been assigned to chromosome 4 in 1983 in one of the first successful linkage analyses using polymorphic DNA markers in humans,^2^ but it took another ten years to pinpoint the gene and determine the mutation. The long lag was largely because this research was conducted before the human genome was mapped, and was the culmination of a painstaking process involving repeatedly refining the location of the gene, based on locating markers and cloning transcripts from the genome across six independent laboratories. The nature of the genetic mutation—an expanded CAG repeat sequence—was also instrumental in the resolution of this detective story. Expanded repeats in DNA had already been associated with several diseases that had features in common with HD, such as genetic anticipation, including fragile X syndrome,^3^ spinal and bulbar muscular atrophy,^4^ and myotonic dystrophy.^5^, ^6^, ^7^ This meant the HD‐CRG were actively looking for length mutations that segregated with disease that might indicate the presence of an expanding repeat tract. As we enter a new phase of HD research, with the advent of trials of potential disease‐modifying treatments, it seems a good time to reflect on the legacy of the HD‐CRG publication.

**FIG. 1:**
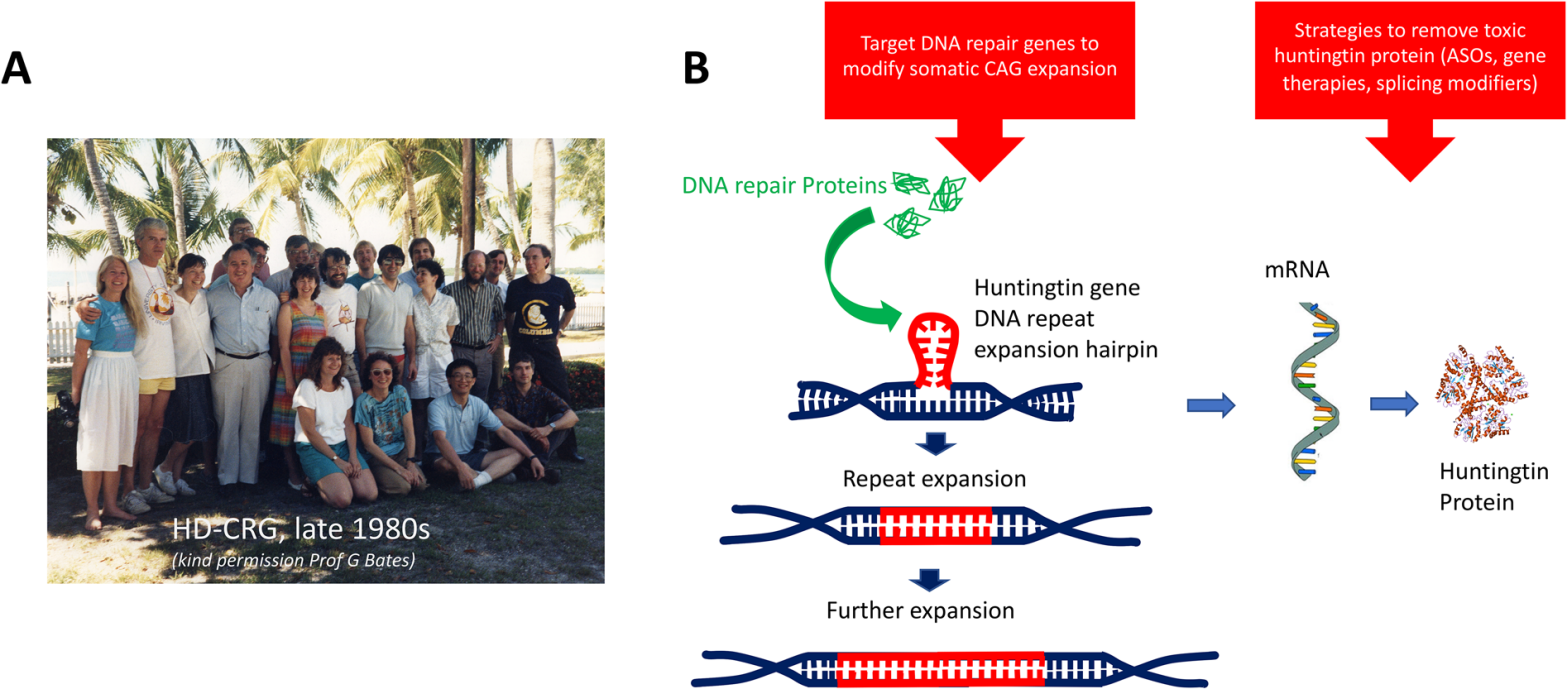
(A) Members of the Huntington's Disease Collaborative Research Group (HD‐CRG), taken at a workshop in Islamorada in the late 1980s (*kindly supplied by Professor G Bates*). (B) The discovery of the gene for HD has led to multiple potential disease‐modifying targets, such as targeting DNA repair proteins with the aim of reducing CAG expansion in the brain, and strategies to reduce levels of huntingtin protein, which aims to reduce the toxic effects of the mutant protein. Fig1 A supplied by Professor Gillian Bates, UCL, London UK.

Understanding the precise nature of the mutation started to throw light on a number of important aspects of disease phenotype. In the HD‐CRG paper, the authors commented on a possible link between CAG repeat number and age of onset, noting the very long repeats seen in juvenile cases. They also noted instability of the repeat when transmitted to the next generation as underlying the phenomenon of genetic anticipation in which subsequent generations can have progressively earlier disease onsets, and demonstrated the link to paternal transmission, with the longest expansions see in juvenile cases where transmission occurred through the paternal line. It raised the possibility of de novo mutations, noting inter‐generational expansion from an allele at the upper end of the normal range, into the disease range in two families in which new mutations had been assumed to have arisen.

The HD‐CRG paper was based on data from 75 HD families from a range of ethnic backgrounds, particularly the large Venezuelan kindreds: it should be recognized that collecting these families was a herculean task that took many years.^8^ It reported that the Huntington's gene (*HTT*) was large and encoded a previously undescribed protein, which they named huntingtin (HTT), and which was widely expressed in many body tissues despite the major impact of HD being in specific regions of the brain. The expanded CAG repeat was located in exon 1 of a 67 exon transcript. Follow up studies established that age at onset of HD was significantly correlated with the length of the CAG tract in *HTT*.^9^, ^10^, ^11^ Recognizing this relationship has helped to accelerate our understanding of how the molecular genetics relates to key aspects of disease phenotype.

The publication of the gene sequence was rapidly followed by the availability of a genetic test which revolutionized preclinical testing by eliminating the need for complicated linkage analysis and provided near 100% accuracy (full penetrance for expansions of 40 and above, with incomplete penetrance for repeats of 36–39). This has made confirmation of diagnosis in symptomatic individuals easier and also raised awareness of the HD phenocopy conditions, the genetic basis for many of these conditions having now been established. Importantly, the gene test allowed predictive testing of an at‐risk individual, without the need for samples from multiple family members that had previously been a limitation for testing based on linkage analysis. The work done on developing protocols for presymptomatic testing in HD provided a route map for testing in other, less common, disorders.^12^ Knowledge of the mutation also makes uterine and pre‐implantation testing feasible; although the uptake of these options remain low,^13^ they can be life‐changing for some families by removing the risk of HD in the next generation.

The value of being able to diagnose the condition with certainty in life also has profound implications for clinical research. The emergence of a reliable genetic test underpinned a number of important large longitudinal observational studies, starting with the North American's Huntington's Study group's Cooperative Huntington Observational Research Trial (COHORT),^14^ followed by the European HD network REGISTRY study,^15^ both of which were superseded by Enroll‐HD.^16^ Enroll‐HD is a global multicenter study with clinical sites in Europe, North America, Australasia and Latin America that provides systematic longitudinal clinical phenotype assessment and a linked biobank for gene positive individuals across all stages of HD (ranging from individuals with no symptoms and no clinical indicators of disease through to those with clear clinical signs). There are now over 20,000 participants in Enroll‐HD and, along with the data collected through REGISTRY and COHORT, it has provided data and biosamples to address a wide range of questions about the clinical phenotype and the biology of HD, in addition to providing important data for developing biomarkers, clinical outcome measures and working out disease‐staging parameters for clinical trials. Collecting large numbers of participants with a rare disease requires international cooperation and multicenter studies, both of which would likely have been too difficult and too expensive in the absence of a readily available genetic test.

The availability of a genetic test, and knowledge that the mutant gene is fully penetrant, had an even more profound effect on the capacity to study human HD before the onset of overt disease, by allowing the identification of individuals carrying the gene before they develop clinical disease. In addition to numerous smaller studies of premanifest individuals, there have been two well‐powered longitudinal premanifest studies; PREDICT‐HD^17^ and TRACK‐HD.^18^ PREDICT‐HD commenced in 2001 and set out to prospectively characterize clinical, neurobiological, and behavioral markers occurring before clinical diagnosis of HD. Less than a decade later PREDICT‐HD was followed by Track‐HD which also aimed to detect clinical and biological markers of HD progression, but with a focus on enabling power calculations and defining the most powerful outcome measures for future trials in participants with very early disease or before formal diagnosis of disease onset. It has also been possible to establish a cohort of “far from onset” young premanifest HD gene carriers, which is starting to identify the very earliest brain changes in HD, more than 20 years before clinical diagnosis, and will be invaluable in defining the earliest stages at which intervention with disease‐modifying strategies may be useful.^19^, ^20^

The discovery of the gene also enabled the generation of genetically accurate models in cells and animals. Models have been generated in species from yeast through to large animals and many of these models have offered useful insights into the pathophysiology of HD.^21^, ^22^, ^23^, ^24^ Most widely used have been mouse models, the first of which, the R6 series, were generated by Gillian Bates.^25^ Her models provided the seminal observation that the HTT protein containing an expanded polyglutamine tract formed the characteristic pathological hallmark of insoluble cellular aggregates in mouse brain, leading to their detection in human brain^26^—although in fact these had been previously observed in human HD brain without their importance being understood.^27^ The R6 model series was generated using a human *HTT* exon 1 transgene, with expansions of 115–150 CAG and ~1000 bp of 5′ sequence, expressed from the human promoter. They represent one of many transgenic models with truncated fragments of *HTT* expressed from various promoters.

Transgenic models containing full length HTT either as a cDNA or the full human sequence in an artificial chromosome and knock‐in models with human‐mouse chimaeric sequences of various types have also been generated. Such models have been instrumental in both understanding the biology of HD and in the search for novel therapeutics.^28^ Most recently, human stem cells derived directly from patients have been developed yielding further insights into HD pathogenesis.^29^, ^30^, ^31^

The HD‐CRG paper also discussed two key areas of understanding which have underpinned disease‐modifier research that are currently in clinical translation. The first of these is huntingtin lowering therapies (Fig. 1B). The paper was able to confirm the previous observation that homozygotes for the disease allele did not differ clinically from heterozygotes, suggesting that HD results from a gain‐of‐function mutation. They considered gain of function mechanisms “in which either the mRNA product or the protein product of the disease allele would have some new property or would be expressed inappropriately” and suggested that the expanded trinucleotide repeats could be translated to an increased poly‐glutamine stretch near the N‐terminus—something that has since been proven to be the case. Although loss of function mechanisms almost certainly play some role in the pathophysiology of HD,^32^ the concept of the mutant protein having gain‐of‐function toxic properties has underpinned the current predominant therapeutic strategy of huntingtin lowering; aiming to reduce the burden of toxic huntingtin protein. Huntingtin lowering in animal models has been shown very clearly to reduce the disease load in the brain and improve function in transgenic HD animals.^33^, ^34^ Various huntingtin lowering strategies are currently in, or close to, clinical trials. These use multiple mechanistic approaches including ASOs interfering with the translation of huntingtin mRNA, delivered into the CSF,^35^ microRNA delivered directly into the striatum using viral vectors, with DNA editing and agents interfering with mRNA splicing currently approaching clinical trial.^36^

A second disease modifying approach that depends on understanding the genetics is that of targeting post‐mitotic expansion of the CAG repeat in the brain (Fig. 1B). The large observational and prospective studies of Predict, Registry and Enroll‐HD have provided large numbers of participants' DNA and matched clinical data for genome‐wide association studies (GWAS) aimed at detecting genetic modifiers of HD. As was outlined by the HD‐CRG paper, as well as being unstable when transmitted to the next generation, the HD trinucleotide repeat is unstable in the absence of recombination or replication. It has since been demonstrated that CAG repeat number continues to increase in certain tissues, in particular the striatum; and the greater the rate of expansion, the lower the age at disease onset.^37^ Identification of the gene and its mutation allowed further genetic research looking for modifiers of age at onset of disease, which has now identified that DNA repair is central in this process, flagged previously in a number of animal studies but not known to be central in human disease onset.^38^ This raises the exciting possibility that targeting DNA repair proteins could reduce *HTT* CAG expansion and may be capable of slowing the disease progression.

Finally, but critically, clinical trials are much more powerful when a condition can be diagnosed with certainty using a reliable and straightforward test, as has been available for HD since 1994. This is especially important for a rare disease where multicenter studies are necessary. Over the last few years, trials of potential disease modifiers of HD have gradually moved to recruiting patients who are earlier in the disease course, and it is likely that this will soon extend to recruitment of patients prior to the onset of motor symptoms; something that would have been extremely difficult, if not impossible, prior to identification of the gene. In summary, the earlier painstaking work of the teams involved in identifying the HD gene have set in motion a whole field of discovery, and hopefully this will be eventually rewarded by identification of a disease‐modifying treatment for this devasting condition.

## Author Roles

1) Research project: A. Conception, B. Organization, C. Execution; 2) Statistical Analysis: A. Design, B. Execution, C. Review and Critique; 3) Manuscript: A. Writing of the first draft, B. Review and Critique.

AER: 3A, 3B

LJ: 3A, 3B

## Disclosures

### Ethical Compliance Statement

The authors confirm that approval of an institutional review board was not required for this work. The authors confirm that patient consent was not required for this work. We confirm that we have read the Journal's position on issues involved in ethical publication and affirm that this work is consistent with those guidelines.

### Funding Sources and Conflicts of Interest

AER is supported through funding from UKRI (MR/T033428/1) and Health and Care research Wales and Horizon 2020. LJ is supported by CHDI, LoQus23 Therapeutics and the MRC (UKDRI studentship). AER and LJ serve on the European Huntington's disease network executive committee. AR and LJ hold renumerated positions on several pharmaceutical industry scientific advisory boards, as declared below.

### Financial Disclosures for the Previous 12 months

AER is Chair of the European Huntington's Disease Network, European co‐PI for the PROOF‐HD trial, and has served on Scientific advisory boards for Roche, Wave pharmaceuticals and Triplet Therapeutics. LJ is a member of the scientific advisory boards of Triplet Therapeutics and LoQus23 Therapeutics.
